# Management of Dehisced Wounds With Exposed Spongiosa With Modern Dressings: A Case Report

**DOI:** 10.7759/cureus.91841

**Published:** 2025-09-08

**Authors:** Davor Jurisic, Bojan Miletic, Marin Marinovic, Tanja Batinac, Goran Slivsek

**Affiliations:** 1 Department of Plastic and Reconstructive Surgery, Clinical Hospital Center Rijeka, Rijeka, HRV; 2 Department of Internal Medicine, General Hospital Ogulin, Ogulin, HRV; 3 Department of Clinical Medical Science, Faculty of Health Studies, University of Rijeka, Rijeka, HRV; 4 Department of Traumatology and Orthopedic Surgery, Clinical Hospital Center Rijeka, Rijeka, HRV; 5 Department of Dermatology, Clinical Hospital Center Rijeka, Rijeka, HRV; 6 Department of Integrated and Palliative Medicine, Clinical Hospital Center Rijeka, Rijeka, HRV

**Keywords:** chronic wound care, modern dressing healing, plastic surgery, wound dehiscence, wound therapy

## Abstract

In this case report, we present the management of a complex wound with an exposed spongiotic sacral bone following surgical dehiscence after bilateral sacroiliac fixation in a 27-year-old patient. Surgical reconstruction was not an option due to the patient's comorbidities. The wound was successfully treated with a combination of MedCu (MedCu Technologies, Herzliya, Israel) and Vacutex (Protex Healthcare, Roeselare, Belgium). A rapid formation of granulation tissue was observed within 10 days, which covered the bone within a few days and led to complete wound closure after a total of four months of treatment. This case highlights the value of advanced wound care technologies as an important alternative for patients who are unable or unwilling to undergo further surgery. It demonstrates that such approaches can facilitate effective wound healing, minimise complications, and significantly improve patients' comfort and quality of life without exposing them to the risks associated with surgery.

## Introduction

Postoperative sacral wounds represent a significant clinical challenge due to their complexity, high risk of complications, and demanding treatment. Postoperative wound infections occur in around 0.5-3% of surgical patients and are associated with a longer hospital stay, additional procedures, and a higher mortality rate [1]. In the sacral region, wound dehiscence with exposed bone is a particularly severe complication, with reported infection and dehiscence rates of 13.2 % and 16.0 %, respectively, as reported by Li et al. [2]. Such wounds often require prolonged hospitalisation and complex reconstructive procedures. The treatment of a sacral wound with exposed cancellous bone, infection, and necrosis is an extremely complex medical challenge. Although surgery is often the quickest and most definitive solution to this problem, it is not suitable for all patients and requires a sophisticated approach based on modern wound care principles, including advanced dressings and optimised local tissue support. Advanced age, significant comorbidities, a weakened general condition, or an increased surgical risk may contraindicate further surgery [3,4]. In such cases, alternative therapeutic strategies become necessary, focusing on wound stabilisation, the prevention or treatment of infection, and the stimulation of granulation tissue formation through modern, non-invasive techniques, such as advanced wound dressings [5]. Modern dressings have been shown to create an optimal wound healing environment by maintaining moisture balance, managing exudate, reducing bacterial load, and protecting against external contamination [6]. Certain dressings also contain bioactive components, including antimicrobial agents, which accelerate tissue repair and reduce the risk of complications [7]. Despite the availability of several advanced dressing options, there is limited evidence specifically on the combined use of MedCu (MedCu Technologies, Herzliya, Israel) and Vacutex (Protex Healthcare, Roeselare, Belgium) in the treatment of dehisced wounds with exposed cancellous bone. Their synergistic properties - MedCu for antimicrobial activity and Vacutex for optimised wound exudate management - may offer advantages over conventional advanced dressings, particularly in high-risk patients who cannot undergo surgery.

This case report fills a gap in the literature by providing detailed clinical evidence for the effective use of these two specialised dressings in a complex wound scenario. Using the case presented, we demonstrate that when applied correctly and to professional standards, these modern dressings can achieve results comparable to invasive surgical techniques while minimising the risks associated with surgery. In the reported case, granulation tissue formation was observed as early as 10 days after initiation of therapy, with complete wound closure achieved within four months of continuous application. This suggests that this structured, non-surgical, two-phase protocol (copper oxide impregnated dressings followed by a rapid capillary action dressing (RCADs)), which results in rapid granulation over exposed bone and subsequent closure, is a viable, evidence-based alternative for similar high-risk patients, and could expand therapeutic options beyond traditional surgical or easy-to-use procedures.

## Case presentation

We present the case of a 27-year-old female patient who suffered multiple traumatic injuries after falling from a window. The patient suffered multiple fractures of the hip, pelvis, sacrum, and lumbar spine. She also had fractures of the feet, lower leg, thigh, cervical spine, and ribs. In addition, a subdural haemorrhage, a heart contusion, a pneumothorax, and a pneumomediastinum were diagnosed. The patient was also diagnosed with anorexia and recurrent depressive disorder with psychotic symptoms.

**Figure 1:**
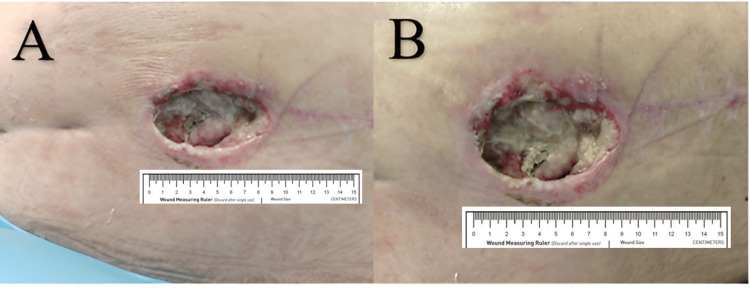
Dehiscent wound with infected tissue (A and B)

In addition to an intensive conservative interdisciplinary approach, the patient required complex surgical interventions for fractures of the legs, hip, and sacrum. Postoperatively, the patient developed a wound infection that led to a dehiscence of the mediosacral surgical site measuring 9 × 5 cm. The dehiscence resulted in the exposure of sacral cancellous bone, along with infected, fibrotic, and necrotic tissue (Figure 1).

**Figure 2:**
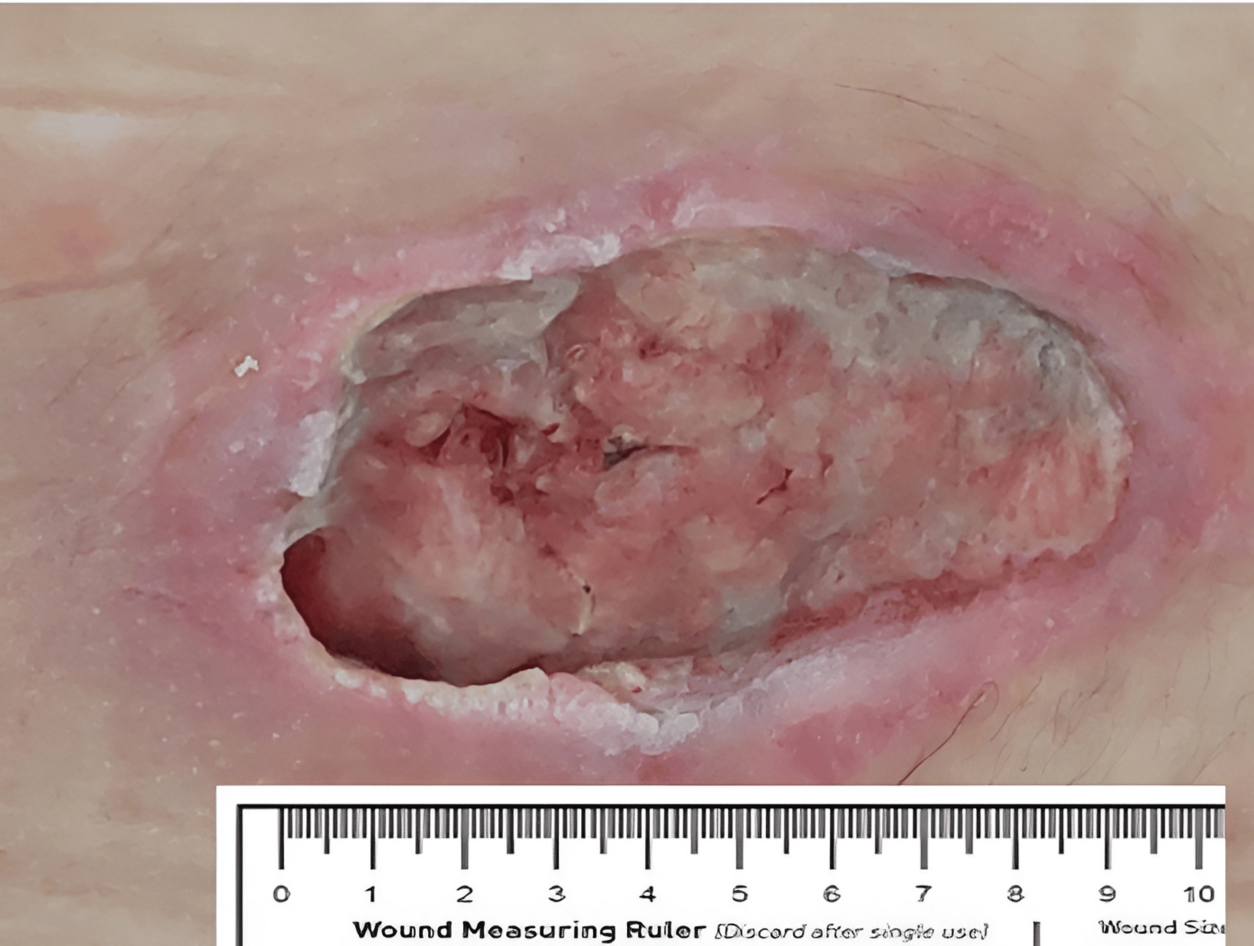
Wound after 10 days of treatment with MedCu shows granulation tissue

The patient developed a urinary infection (*Enterobacter hormaechei*), which was treated with piperacillin-tazobactam. *Enterococcus faecalis* was isolated from the wound swab, which was further treated with daily local dressings in addition to antibiotic therapy. In view of the patient's mental illness, underlying anorexia with associated hypoalbuminaemia, lack of subcutaneous fat and muscle, and inability to achieve adequate nutritional support, mainly due to her limited cooperation and engagement related to her mental state, surgical treatment of the dehiscent mediosacral wound was contraindicated. It was therefore decided to initiate treatment with copper oxide-impregnated dressings - MedCu. After 10 days of wound treatment with MedCu dressings, the wound bed was covered with granulation tissue that completely covered the previously exposed bone. The wound edges showed epithelialization (Figure 2).

Resolution of the infection was confirmed by normalised laboratory parameters (C-reactive protein, white blood cell count), absence of fever, and a normal microbiological wound swab, as shown in Table 1.

**Table 1:** Laboratory findings of the patient

Parameter	Day 0	Day 14	Day 15	Day 24	Two months	Reference range
Erythrocyte sedimentation rate	61	95	100	-	-	4-24 mm/hour
Hemoglobin	123	117	109	122	126	119-157 g/L
Platelets	472	299	635	683	310	158-424 × 10^9^/L
Leukocytes	9.5	11.0	13.6	7.4	7.2	3.4-9.7 × 10^9^/L
C-reactive protein	-	123	240	30.7	12.1	0-5.0 mg/L
Procalcitonin	-	0.10	0.26	-	-	<0.5 µg/L
Albumin	40	36	-	40	43	41-51 g/L
Microbiological findings						
Blood cultures	Day 14, Day 15: Bacteriologically sterile					
Urine cultures	-	Day 14: *Enterobacter hormaechei*		Day 24: Bacteriologically sterile		
Wound swab	-	Day 14: *Enterococcus faecalis*		After two months: *Staphylococcus haemolyticus*, *Staphylococcus capitis*		

**Figure 3:**
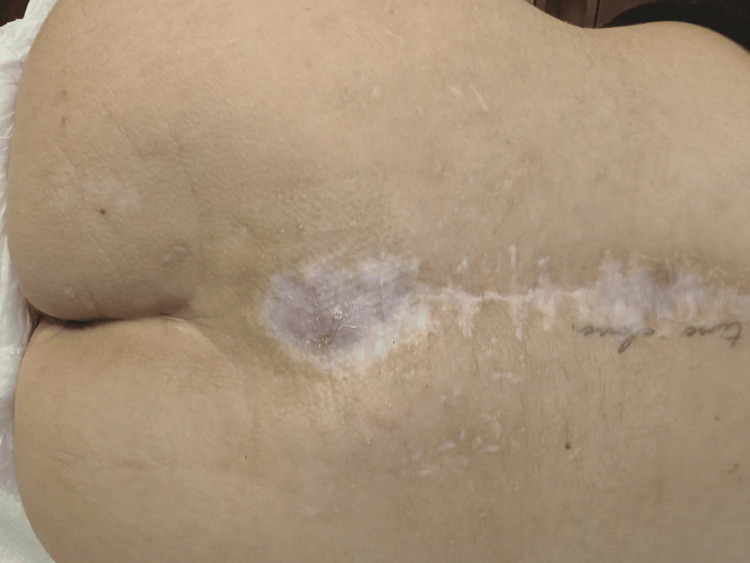
Final result after four months of treatment

Wound therapy with MedCu dressings was carried out over two months, with the dressings being changed every two days. Staphylococcus haemolyticus and Staphylococcus capitis were isolated from the wound. This finding was interpreted as colonisation without any further increase in inflammatory laboratory parameters. The radiological findings during the entire course of treatment did not indicate the development of osteomyelitis. As a result of the ongoing polytrauma, the patient was predominantly immobilised and bedridden, which enabled regular wound care and ensured optimal conditions for dressing changes. Mobilisation was limited to physiotherapy sessions, during which the patient was able to walk for up to one hour per day under supervision. Wound exudation remained minimal throughout the treatment. After the patient slowly became more physically active, the secretion of the wound increased. Once the infection was brought under control, granulation tissue formed, and the exudate was persistent, the therapeutic goal shifted to effective exudate management. At this point, capillary-active exudate management without suction was introduced to optimise fluid transport, maintain a moist environment, and support progressive wound contraction. Vacutex was applied directly to the wound bed, ensuring full contact with the exudative surface. A double layer was used for the first ten days, after which it was reduced to a single layer as the amount of exudate decreased. The dressing was changed every 24 hours. It was held in place with sterile gauze and medical tape. No special skin care was performed. By implementing the above therapy, epithelialization continued to progress. The entire treatment of the patient lasted four months and resulted in complete wound healing (Figure 3).

The clinical course and interventions are presented in Table 2.

**Table 2:** Timeline of clinical course and interventions

Time point	Clinical status and wound findings	Intervention/treatment	Photo documentation
Day 0	Polytrauma following a fall: initial stabilization and intensive care	Stabilization, fracture fixation, supportive care	-
Week 2	Development of a mediosacral wound dehiscence with bone exposure, a urinary tract infection (*Enterobacter hormaechei*), and a local wound infection (*Enterococcus faecalis*), accompanied by malnutrition and hypoalbuminemia.	Surgical treatment contraindicated, initiation of local wound treatment with MedCu copper oxide dressings, treatment with piperacillin-tazobactam	Yes (baseline wound)
Day 10 after therapy starts	Rapid granulation tissue formation observed; bone covered	Continuation of the MedCu regimen	Yes
Month 2	Significant wound contraction, infection controlled; no further necrosis	Continuation of the MedCu regimen; Patient mobilized	-
Month 3	Increased secretion of the wound	Start with Vacutex. As part of the rehabilitation programme, one hour of walking with the support of a physiotherapist (full load on the left leg and 10 kg load on the right leg, with weekly increases of 3-5 kg).	-
Month 4	Complete wound closure	Standard skin care; patient independently mobile	Yes (final result)

## Discussion

The treatment of complex wounds, particularly those with exposed bone and infection, remains a significant challenge, especially when surgical closure is limited or contraindicated, as in our 27-year-old patient with severe polytrauma, multiple comorbidities, and a severe psychiatric disorder. Given the patient’s poor general condition, severe malnutrition, persistent infection, and contraindication to surgical treatment, local wound care remained the only viable option. In this context, the use of modern wound dressings, MedCu and Vacutex, proved to be an effective therapeutic approach.

Modern wound dressings are advanced wound care products that maintain a moist wound environment, promote autolytic debridement, protect against bacterial infiltration, and support tissue regeneration. They are available in a variety of forms, such as hydrogels, foams, vapour-permeable films, alginates, and silicone meshes, each tailored to specific wound types and stages [8]. Modern dressings are now a standard therapeutic approach for the treatment of wounds that heal with or without skin defects. These dressings provide optimal physico-chemical conditions for wound healing. They create an additional barrier against microorganisms, thus supporting the body’s natural defence mechanisms and reducing the risk of infection. They not only create a moist, occlusive environment that accelerates healing and promotes autolytic debridement, but also reduce wound pain and offer patients a form of atraumatic protection [9,10].

The application of modern dressings can be customised to the stage and type of wound, allowing for a more targeted and effective therapeutic approach. Modern dressings impregnated with copper oxide (MedCu dressings) create an optimal moist environment for healing, while copper has antimicrobial properties, stimulates angiogenesis, and supports the formation of the extracellular matrix, thereby accelerating granulation tissue formation and epithelialisation [11,12]. Histological studies have shown that copper stimulates the proliferation of keratinocytes and fibroblasts and promotes the formation of new blood vessels, which is crucial for the healing of wounds with deep defects [13]. Compared to silver-impregnated dressings, copper is equally effective but with potentially less cytotoxicity and better tolerability [11]. However, as the patient gradually mobilised, the increasing wound exudate necessitated a change in treatment. Vacutex dressings, which create negative pressure through mechanical capillary action, allowed therapy to continue without the need for a fixed device for negative pressure wound therapy (NPWT), also known as vacuum-assisted closure (VAC) [14,15]. Vacutex works on a similar principle but without an electrical device, allowing the patient greater mobility and therefore more effective physical therapy and faster rehabilitation [16]. In our case, the Vacutex helped to maintain the progress made in wound healing, reduce the risk of further complications, and accelerate overall recovery. These types of modern dressings, known as RCADs, are effective when NPWT is not sufficiently effective, particularly in the treatment of sloughy, devitalised, and exudative wounds [10]. Janssen also emphasises the promising results of RCAD as a replacement for NPWT or as a possible “step-down” model: start with NPWT and switch to RCAD when the wound shows signs of healing [17].

## Conclusions

The treatment of a complex, infected wound with exposed bone in this critically ill patient illustrates the potential role of modern wound dressings as a non-surgical alternative when surgical options are not feasible. In this case, the MedCu and Vacutex dressings appeared to provide benefit through their antimicrobial, angiogenic, and mechanical debridement properties, supporting wound healing and enabling early rehabilitation. While these results are encouraging, it must be emphasised that this is a single case report, and no conclusions can be drawn regarding overall efficacy or therapeutic superiority. This report highlights a potential treatment option in selected patients and emphasises the need for further research to assess the wider clinical benefits of these dressings.
